# Coronary artery disease: ‘gout’ in the artery?

**DOI:** 10.1093/eurheartj/ehab276

**Published:** 2021-05-29

**Authors:** Timo E Strandberg, Petri T Kovanen

**Affiliations:** 1 University of Helsinki, Clinicum, and Helsinki University Hospital, Haartmaninkatu 4, PO Box 340, FIN-00029 Helsinki, Finland; 2 University of Oulu, Center for Life Course Health Research, Aapistie 5, 90014 Oulu, Finland; 3 Wihuri Research Institute, Haartmaninkatu 8, 00290 Helsinki, Finland

## Abstract

**Figure:**
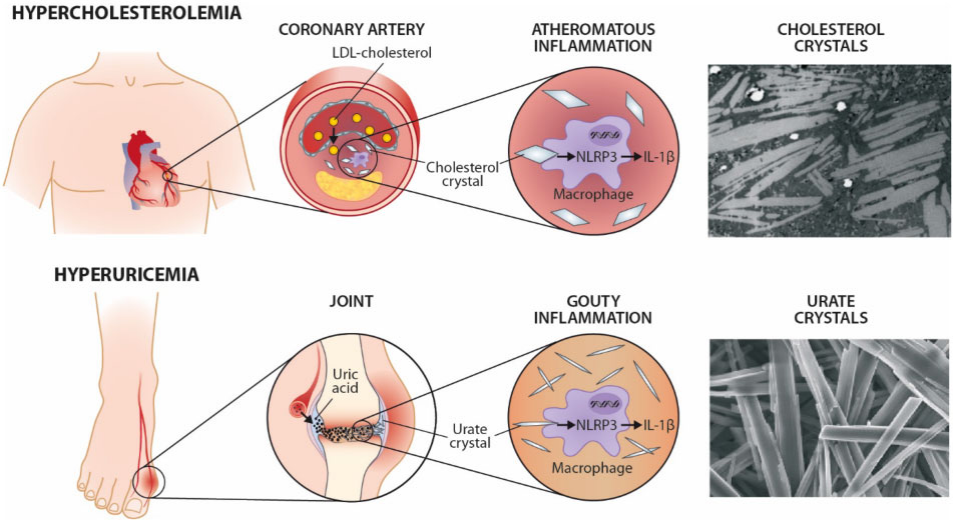
Atherosclerotic cardiovascular disease and gout through a ‘crystal lens’. We thank Prof. Zhou for the images of urate crystals (Molloy R.G.E., Sun W., Chen J., Zhou W. Structure and cleavage of monosodium urate monohydrate crystals. *Chemical Communications*, 2019, 5). Transmission electron micrograph (courtesy of Satu Lehti and Katariina Öörni, Helsinki, Finland) of cholesterol crystals is from an endarterectomized human carotid atherosclerotic lesion (Helsinki Carotid Endarterectomy Study 2, HeCES2). The micrograph was taken at the Electron Microscopy Unit, Institute of Biotechnology, University of Helsinki, Finland.

Atherosclerotic cardiovascular disease and gout through a ‘crystal lens’. We thank Prof. Zhou for the images of urate crystals (Molloy R.G.E., Sun W., Chen J., Zhou W. Structure and cleavage of monosodium urate monohydrate crystals. *Chemical Communications*, 2019, 5). Transmission electron micrograph (courtesy of Satu Lehti and Katariina Öörni, Helsinki, Finland) of cholesterol crystals is from an endarterectomized human carotid atherosclerotic lesion (Helsinki Carotid Endarterectomy Study 2, HeCES2). The micrograph was taken at the Electron Microscopy Unit, Institute of Biotechnology, University of Helsinki, Finland.

In medicine, it is not uncommon that phenotypically quite different diseases end up having similar pathways in their pathogenesis. In this vein, recent trials with canakinumab (Canakinumab Anti-inflammatory Thrombosis Outcome Study, CANTOS^1^), and with colchicine—most convincingly the Colchicine Cardiovascular Outcomes Trial (COLCOT^2^) and the Low Dose Colchicine 2 trial (LoDoCo2)^3^—conceptually combine two diseases, gout and atherosclerotic cardiovascular disease (ASCVD), intuitively considered to be very apart from each other.

While the clinical and pathogenetic characteristics of ASCVD have received much attention during the past decades, the characteristics of gout may be less familiar to cardiologists. The primary tissue targets in these two diseases, i.e. joints and kidneys in gout, and arteries in ASCVD are completely different. Accordingly, we do not intend to claim that hyperuricaemia, the root cause of gout, would affect the developing ASCVD. Of note, although numerous studies have suggested that uric acid may play roles in ASCVD, the causal connection is still controversial.^4^ Likewise, there is no indication that hypercholesterolaemia as such would be involved in gout. However, there are interesting parallels between their basic pathologies, acute treatments, and management of their risk factors, hyperuricaemia and hypercholesterolaemia, respectively. This being a Viewpoint, we take the freedom to assess basic pathology through a ‘crystal lens’,^5^^,^^6^ and intentionally give less attention to the roles of the myriad of other factors related to the multifactorial pathogenesis of ASCVD.

In brief, gout is becoming a more widespread type of chronic inflammatory arthritis, affecting up to 4–9% of adults in some Western and Asian societies.^7^ Its classic and dramatic presentation is characterized by recurrent attacks (flares) of red, painful, hot, and swollen joints, especially in the base of the big toe, knee, and less commonly in other joints. Like cholesterol, uric acid is a natural product being the metabolic breakdown of purine nucleotides, either from endogenous or dietary sources. It is mainly excreted via kidneys as a normal component of urine. In the body, uric acid presents as such, or as ions and salts (urates). In gout, the normal homeostasis of uric acid metabolism is disturbed—most commonly due to reduced excretion—leading to its high circulating concentrations (hyperuricaemia), which may then lead to acute episodes of joint inflammation and kidney stones. This is because hyperuricaemia predisposes to high uric acid concentrations also in tissues, thereby increasing the possibility of local formation of monosodium urate crystals. The crystals trigger primarily in mononuclear phagocytes, such as macrophages, an inflammatory response which is mediated by the nucleotide-binding oligomerization domain-(Nod-)like receptor3 (NLRP3) inflammasome, and leads to caspase-1-dependent conversion of pro-interleukin (IL) 1β into mature IL-1β that may be secreted by the cells. On the cell surfaces of several cell types, the secreted IL-1β binds to IL-1 receptors, which then activate a signalling pathway leading to the activation of the transcription factor nuclear factor-kappa B. Ultimately, this inflammatory cascade leads to the release of various inflammatory cytokines, chemotactic factors, lysosomal enzymes, eicosanoids, and reactive oxygen species, which affect a host of inflammatory cells, prominently including various mononuclear phagocytes, and also neutrophils and mast cells.

However, the crystals alone do not seem to be able to trigger the flare; a second signal is also required.^7^ Free fatty acids can be this signal, and their involvement helps to explain the classic picture of an acute gout attack after a heavy meal and alcohol consumption. In the absence of any treatment, the resolution of inflammation takes place as an active and timely process, which ultimately leads to the restoration of homeostasis.

It is noteworthy that 85–90% of people with hyperuricaemia never suffer from acute flares, just as not all persons with hypercholesterolaemia experience a heart attack. However, if the solubility of urate is exceeded, crystals can nevertheless trigger a chronic inflammatory response presenting as urate-containing nodules, tophi, which protrude from the skin, accumulate in various tissues, and cause bone erosions. This situation closely resembles familial hypercholesterolaemia in which very high cholesterol concentrations cause the formation of cholesterol-containing xanthomatous skin and tendon nodules.

In ASCVD, the root cause is hypercholesterolaemia, which is pathophysiologically relevant at LDL cholesterol level >1.4 mmol/L (55 mg/dL) and predisposes to the harmful intrusion of LDL particles into the arterial wall.^8^ In the arterial wall, the LDL particles become oxidized or otherwise modified and then induce an immunological and inflammatory response, which, together with local cholesterol accumulation becomes the nidus to the development of an atherosclerotic plaque. An interesting parallel to gout is that like urate in the joint, free cholesterol relieved from LDL particles may crystallize in the arterial wall. Mechanistically then, the connection between cholesterol and the inflammatory component of ASCVD can be partly explained by the fact that, like cellular uptake of the monosodium urate crystals, also the uptake of cholesterol crystals activates the NLRP3 inflammasome and induces IL-1β secretion with ensuing local inflammation.^5^^,^^6^ Whether an additional activation signal is needed, e.g. via uptake of LDL-derived oxysterols by the macrophages, is currently debated.^9^ Nevertheless, the inflammatory process predisposes to the formation of vulnerable plaques susceptible to rupture and ensuing acute cardiovascular events. This may be seen as analogous to a gout flare.

From a treatment point of view, recent evidence tells us that both gout and ASCVD ‘flares’ can be counteracted by the same therapies. In CANTOS, a fully human anti-IL-1β neutralizing monoclonal antibody, canakinumab, reduced residual ASCVD risk in patients already on maximal lipid-lowering therapy.^1^ But canakinumab also reduced gout attacks.^10^

Similarly, in COLCOT^2^ and LoDoCo2,^3^ colchicine, an ancient medication historically known to be effective in acute gout, reduced ASCVD endpoints on top of lipid-lowering therapy. While canakinumab selectively blocks IL-1β, colchicine, as a natural product, has a wider range of anti-inflammatory effects, also upstream of the NLRP3.^11^^,^^12^

We have earlier compared the effects of intensification of LDL cholesterol lowering, i.e. when adding proprotein convertase subtilisin/kexin-type 9 inhibitor (PCSK9i) therapy on top of statin therapy to those achieved when adding an anti-inflammatory drug on top of statin therapy.^13^ This comparison indicated that inflammation inhibition is effective on top of very aggressive LDL lowering. Of the CANTOS, COLCOT, and LoDoCo2 participants, almost everyone received concomitant optimal statin therapy (although the levels of blood lipids were not reported in the colchicine trials). In the two PCSK9i trials, ODYSSEY (similar to COLCOT regarding a recent coronary artery disease event with ensuing myocardial ischaemia) and FOURIER (similar to LoDoCo2 regarding the presence of chronic coronary artery disease), practically all patients received statin therapy of at least moderate intensity.^13^ Despite statin treatment, the average LDL cholesterol levels were 92 mg/dL (2.4 mmol/L) at baseline, which is well above the 55 mg/dL (1.4 mmol/L) level considered to protect from the progression of ASCVD.

The primary cardiovascular disease endpoints in both COLCOT and LoDoCo2 started to decline very early after the initiation of colchicine therapy, and they were mainly due to a reduction of myocardial infarction and acute ischaemia-driven revascularization.^2^^,^^3^ Thus, in principle, colchicine administration for coronary artery disease resembled ‘gout therapy’.

Inflammation is involved in various disease processes and, for example in CANTOS,^1^ IL-1β inhibition also reduced the risk of anaemia and knee osteoarthritis. It is of note that despite reducing acute effects and endpoints in gout and ASCVD, neither canakinumab nor colchicine reduces the root cause of gout and ASCVD: plasma uric acid and LDL cholesterol level, respectively. Neither do they, at present, seem suitable for long-term prevention, because of high cost (canakinumab) or possible concerns about overall safety (colchicine).^14^ More research is needed on the latter, and overall on focused therapies upstream of NLRP3 inflammasome. However, if the root cause, i.e. hyperuricaemia or hypercholesterolaemia are kept at bay, it is obvious that there would be no urate crystals to induce flares or cholesterol crystals to contribute to ASCVD. Concerning the marching order of the pathophysiological factors in ASCVD, it is intriguing that in the primitive Tsimane population neither a high infectious burden nor a high C-reactive protein level was associated with ASCVD, most probably because the LDL cholesterol level in this population is very low.^15^ This notion is supported by the crude similarity of benefits achieved by an anti-inflammatory therapy on top of the moderate baseline LDL cholesterol level (82 mg/dL; 2.1 mmol/L) in CANTOS and, alternatively, by a very effective LDL cholesterol lowering down to 40 mg/dL (1.0 mmol/L) involving PCSK9i therapy without the need for additional anti-inflammatory treatment.^1^^,^^13^ However, neither therapy could fully abolish residual risk, the probable explanation for this being a late start of the treatment when considering the normally long latent period before the onset of clinical ASCVD.

Accordingly, there is further parallelism between gout and ASCVD: the necessity of a long-term strategy to prevent endpoints by addressing the root cause of the respective disease to be treated. In gout, a prolonged effective urate-lowering therapy by xanthine oxidase inhibitors like allopurinol or febuxostat, uricosurics, absorption inhibitors, or uricase agents can effectively reduce the frequency of gout attacks because the rate of crystal dissolution is slow and proportional to the degree of the urate-lowering achieved.^7^ In ASCVD, again, a therapy that inhibits hepatic cholesterol synthesis (statin), inhibits intestinal cholesterol absorption (ezetimibe), or prevents the degradation of hepatic LDL receptors (PCSKi), ultimately stimulates the hepatic LDL receptor-mediated uptake of LDL particles, and has been used either alone or in combination to control LDL cholesterol level in the circulation. The efficacy of these therapies for reducing ASCVD endpoints is firmly established.^8^ Although xanthine oxidase inhibitors are implicated to affect surrogate endpoints (blood pressure and endothelial function), their effects on clinical outcomes of ASCVD have not been established.^4^ Inversely, cholesterol lowering is not known to affect uric acid or gout.

## Conclusion

In a ‘system biology perspective’, inflammation and its potential triggers, the crystals, are unifying factors in the pathophysiology of both gout and ASCVD (*Graphical abstract*). Furthermore, therapies to diminish the crystal-induced inflammation with canakinumab or colchicine have been shown to prevent the respective clinical presentations, especially the acute ones. A final common similarity is that the root causes—hyperuricaemia in gout and hypercholesterolaemia in ASCVD—must be effectively addressed to attain the best results for patients in the long term.

### Author’s Contributions

Both authors prepared the paper together.
